# Modified crystallite group method for residual stress analysis of highly textured Cu/Mo nanomultilayers

**DOI:** 10.1107/S1600576726003638

**Published:** 2026-05-20

**Authors:** Jeyun Yeom, Claudia Cancellieri, Amit Sharma, Bastian Rheingans, Xavier Maeder, Gregory Abadias, Jolanta Janczak-Rusch

**Affiliations:** a Laboratory for Joining Technologies and Corrosion EMPA, Swiss Federal Laboratories for Materials Science and Technology Überlandstrasse 129 Dübendorf8600 Switzerland; b Laboratory for Mechanics of Materials & Nanostructures EMPA, Swiss Federal Laboratories for Materials Science and Technology Feuerwerkstrasse 39 Thun3602 Switzerland; c Institut Pprime, Université de Poitiers-CNRS-ENSMA, Boulevard Marie et Pierre Curie, Poitiers Cedex 09, 86073, France; Montanuniversität Leoben, Austria

**Keywords:** Cu/Mo nanomultilayers, X-ray diffraction, residual stress, texture analysis, modified crystallite group method

## Abstract

Residual stress analysis in thin films and nanomultilayers (NMLs) is essential for understanding their mechanical, thermal and functional properties. However, accurate strain measurements in highly textured multilayers obtained by physical vapor deposition techniques can be experimentally challenging, particularly when strong epitaxial relationships and twinning are present. In this paper, texture and residual stresses were investigated for a representative NML system composed of immiscible face-centered cubic (fcc)/body-centered cubic (bcc) elemental layers, namely Cu/Mo NMLs grown on a sapphire (0001) substrate. The experimentally investigated Cu/Mo NML system exhibits a specific fcc Cu {111}||bcc Mo {110}||sapphire (0001) in-plane and out-of-plane texture with growth twinning of Cu and a Nishiyama–Wasserman orientation relationship between Cu and Mo. This combination results in an effective sixfold rotational symmetry in the pole figures. The presence of twinning significantly complicates residual stress determination, as the selection of inappropriate diffraction planes can lead to erroneous results and the failure of standard approaches like the crystallite group method. To address this issue, we have established a theoretical framework and practical methodology to select suitable diffraction planes for the reliable determination of residual stresses in fcc/bcc NMLs with strong in-plane and out-of-plane textures, including the presence of twinning, using X-ray diffraction. The experimental X-ray-diffraction-based analysis is complemented by high-resolution scanning transmission electron microscopy and orientation mapping, providing a comprehensive understanding of the microstructure and crystallographic texture. The presented method can be applied to other multilayer systems and extended to other orientation relationships.

## Abstract 2

We present a modified crystallite group method that enables reliable X-ray-based residual stress analysis in highly textured nanomultilayers exhibiting strong in-plane orientation relationships and growth twinning. By establishing a robust diffraction-plane selection framework and validating it through high-resolution scanning transmission electron microscopy and texture analysis, we provide a broadly applicable methodology for accurate stress evaluation in epitaxial multilayer systems.

## 1. Introduction

Residual stresses in thin multilayered films prepared by magnetron sputtering significantly affect their material properties, thermal behavior and reliability across a wide range of application fields including microelectronics, X-ray optical mirrors and optoelectronics. Residual stresses can be highly detrimental for nanomultilayers (NMLs), *e.g.* for their mechanical properties: high tensile or compressive stress, for instance, may lead to cracking, buckling or delamination of the film (Welzel *et al.*, 2005 ▸). On the other hand, tuning the residual stresses of NMLs is also an effective way to tailor their properties, *e.g.* their thermal stability and conductivity (Lorenzin *et al.*, 2022*a* ▸,*b* ▸). Therefore, analyzing residual stress in NMLs is essential for understanding their microstructural evolution, *e.g.* during heat treatment processes, as well as their performance and reliability in application.

Several experimental techniques are available for measuring residual stresses in thin films. These include curvature measurement, deformation techniques, X-ray diffraction (XRD), neutron diffraction and other specialized methods, which have been successfully applied to a range of thin films and nanomaterials (Labat *et al.*, 2004 ▸; Misra *et al.*, 2000 ▸; Zhang & Misra, 2004 ▸; Aydıner *et al.*, 2007 ▸; Romano-Brandt *et al.*, 2020 ▸; Cordill *et al.*, 2018 ▸; Yeom *et al.*, 2023 ▸) and have been concisely reviewed (Abadias *et al.*, 2018 ▸). Interfacial stress, defined as the elastic deformation energy at the interface (Ruud *et al.*, 1993 ▸; Jaouen *et al.*, 2001 ▸), can be characterized using two complementary approaches: XRD for assessing residual stress within individual layers, and curvature measurements for determining the force exerted on the underlying substrate. This combined methodology offers a comprehensive evaluation, crucial for understanding the structural integrity and performance of NMLs and their various elastic-strain-dependent properties (Druzhinin *et al.*, 2021 ▸; Cammarata & Sieradzki, 1989 ▸; Cammarata, 1994 ▸).

In NMLs, typically fabricated by physical vapor deposition techniques such as sputtering, the pronounced crystallographic texture commonly observed poses a significant challenge for strain measurements. The conventional sin^2^Ψ method, which assumes a random polycrystalline orientation distribution, is therefore not applicable for such systems (Welzel *et al.*, 2005 ▸; Noyan & Cohen, 1987 ▸). In textured materials, the lattice spacing *d* often exhibits a nonlinear dependence on sin^2^Ψ, unlike isotropic materials where a linear relationship is expected. This deviation arises from material anisotropy (*e.g.* elastic constants) and preferred orientation effects, which also lead to broader and weaker diffraction peaks (Kapoor *et al.*, 2002 ▸). To overcome these limitations, the crystallite group method (CGM) approach was developed, providing a more accurate framework for residual stress evaluation in strongly textured samples. The CGM assumes that all crystallites sharing a common crystallographic orientation constitute one crystallite group, which can then be treated as a single crystal for stress analysis (Welzel & Mittemeijer, 2004 ▸; Gergaud *et al.*, 1998 ▸). This approach has been successfully applied to systems such as Cu (111)/W (110) NMLs (Cancellieri *et al.*, 2016 ▸; Lorenzin *et al.*, 2022*a* ▸). Because W is elastically isotropic, any orientation-dependent component of the in-plane strain cancels out. For fcc materials with a {111} texture, the in-plane strain shows no azimuthal (in-plane angular) dependence. In the general case, accurate implementation of the CGM requires careful selection of diffraction planes that reflect the texture symmetries of the sample. Inappropriate plane selection can lead to significant errors and ultimately cause the method to fail, necessitating alternative strategies. Several approaches using a modified CGM method to account for texturing have been reported (Tanaka *et al.*, 1999 ▸, 1998 ▸, 1996 ▸; Yeom *et al.*, 2023 ▸), but these are typically limited to fiber-textured systems and assume equi-biaxial residual stresses (Yeom *et al.*, 2023 ▸; Clemens & Bain, 1992 ▸). Such assumptions are not valid in cases of strong in-plane texture, such as epitaxial or highly textured multilayers, necessitating a different approach for accurate stress evaluation.

In a previous study, the residual stresses in epitaxial Mo (110)/Ni (111) NMLs, with strong in-plane and out-of-plane texture and a Nishiyama–Wasserman (NW) orientation relationship (OR) between Ni and Mo lattices, were successfully analyzed using XRD (Abadias *et al.*, 2010 ▸). However, that work did not provide a detailed rationale or procedure for selecting appropriate diffraction planes, an omission that can lead to inaccurate stress evaluation. This underscores a broader challenge in residual stress analysis of textured NMLs: the need for a systematic and reliable approach to diffraction plane selection that ensures consistent and accurate stress evaluation across different material systems.

To address this issue, the present study investigates Cu (111)/Mo (110) multilayers, which exhibit similar strong texture and twinning characteristics, to develop and demonstrate such a methodology. We establish both a theoretical framework and a practical methodology to select diffraction planes for the accurate determination of the residual stresses in NML systems composed of immiscible face-centered cubic (fcc) and body-centered cubic (bcc) materials, such as the Cu/Mo system. These multilayers often display pronounced in-plane and out-of-plane textures, as well as crystallographic twinning, which complicate residual stress analysis. Using Cu (111)/Mo (110) multilayers deposited on sapphire (0001) as a model system, we combine XRD and high-resolution scanning transmission electron microscopy (HR-STEM) to characterize the texture and microstructure. Residual stresses are then analyzed using a modified CGM, after optimal crystallographic plane selection, thereby overcoming the limitations of conventional CGM in the presence of twinning. Although the analysis is developed for NW-type ORs with twinning, the methodology is broadly applicable to other ORs in textured multilayer systems.

## 2. Experimental procedure

Cu/Mo NMLs were deposited at room temperature on a sapphire (0001) substrate by DC magnetron sputtering in a high-vacuum chamber (pressure ≤ 10^−8^ mbar; AJA Orion 8 system) using Cu (99.99% purity) and Mo (99.95% purity) targets. The substrates were sequentially cleaned with acetone, ethanol and propan-2-ol using ultrasonic agitation for 3 min each, followed by drying in an Ar flow. Depositions were carried out in pure Ar at a working pressure of 0.27 Pa, with a sputtering power of 80 W for both targets. The deposition rates in these conditions were 0.143 and 0.625 nm s^−1^ for Mo and Cu, respectively. The multilayer structure consisted of alternating 10 nm Cu and 10 nm Mo layers (20 nm bilayer period), repeated ten times to achieve a total NML thickness of 200 nm.

Cross-sectional lamellae for transmission electron microscopy (TEM) were prepared by focused ion beam milling (Tescan, Lyra3) utilizing a 30 kV Ga ion beam. To eliminate the damaged surface layer after cutting, a cleaning process was conducted with 5 and 2 kV Ga ion beams sequentially. High-angle annular dark field (HAADF) and bright field (BF) scanning transmission electron microscopy (STEM), as well as BF TEM, were carried out using an aberration-corrected transmission electron microscope (Themis 200 G3, Thermo Fischer) equipped with an energy-dispersive X-ray spectroscopy detector and operated at 200 kV. Orientation imaging of the cross-sectional lamella was conducted using scanning precession electron diffraction (SPED), a technique integrated into the transmission electron microscope (NanoMegas Digital/ASTAR), which enables mapping at nanometre resolution (Portillo *et al.*, 2010 ▸).

A Bruker D8 Discover X-ray diffractometer with a 1D Lynxeye detector was used to determine the texture of the sample and the residual stresses in the NMLs. Diffraction patterns with Cu *K*α radiation were recorded using a Cu anode operated at 40 kV and 40 mA. To assess the texture, pole figures of Cu {111} and Mo {110} reflections were recorded. The tilt angle varied in steps of 3° from 0 to 80°. Additionally, φ scans of Cu {111}, Mo {110} and Mo {321} were used to evaluate the intensity of texture variants. In the present work, ‘out-of-plane texture’ refers to the distribution of crystallographic orientations with respect to the surface normal of the film. In diffraction terms, this describes how strongly a given crystallographic direction (*e.g.* the film growth direction) is aligned with the surface normal and it is probed through measurements as a function of the tilt angle ψ. Conversely, ‘in-plane texture’ describes the azimuthal orientation distribution of the crystallographic axes within the film plane, characterized by rotations around the surface normal (φ dependence) observed in the pole figures or azimuthal scans.

## 3. Results and discussion

### 3.1. Texture analysis of Cu/Mo NMLs

**Figure 1:**
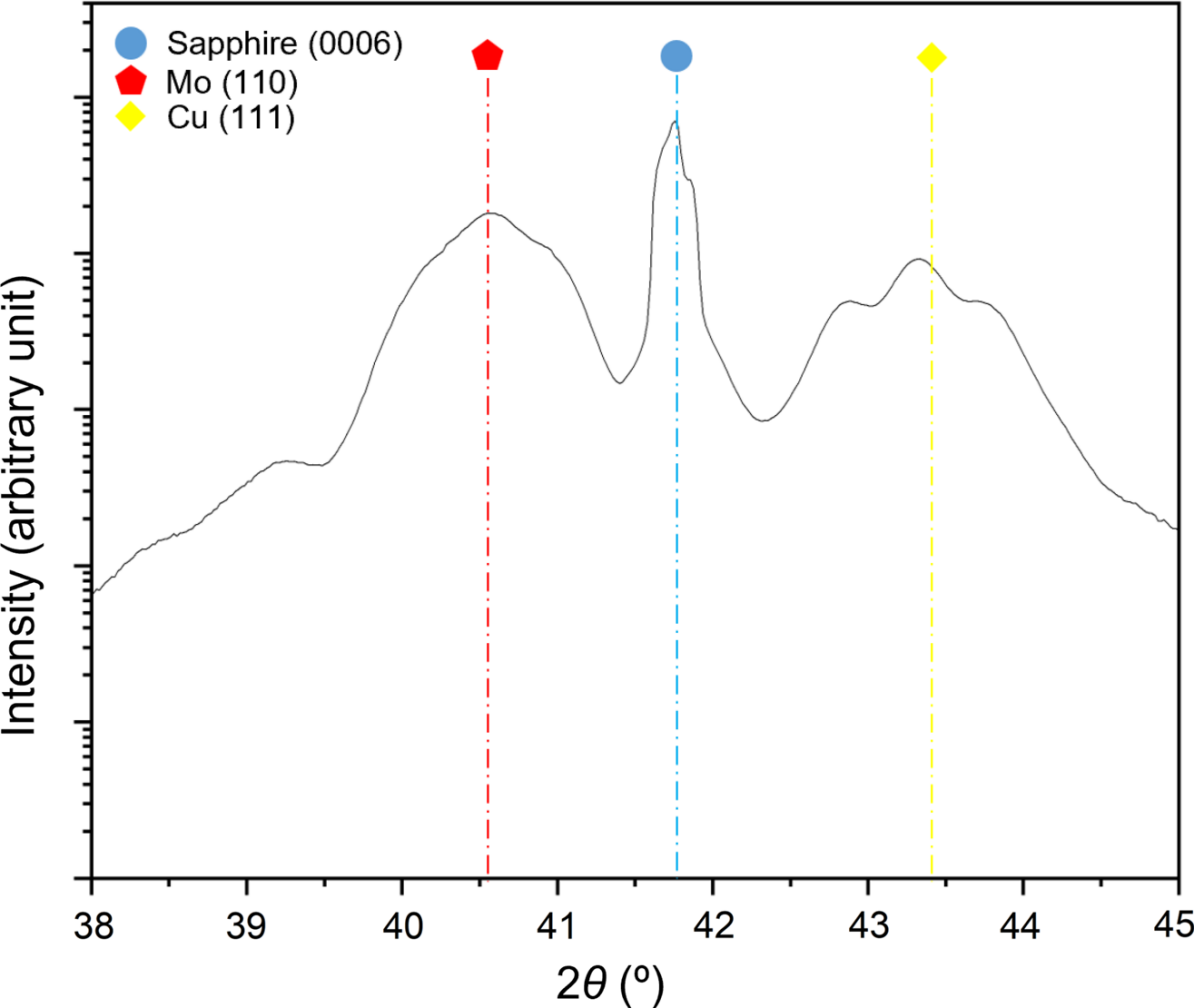
XRD θ–2θ scans (in logarithmic scale) of Cu/Mo NMLs grown on sapphire (0001) substrate.

Fig. 1 ▸ shows the XRD 2θ profile of the Cu/Mo NMLs deposited on sapphire, plotted on a logarithmic scale in the selected range from 38° to 45°. The diffraction pattern displays well-defined satellite peaks of high intensity, characteristic of the superlattice periodicity within the Cu/Mo multilayer structure. These high-angle satellite peaks can be described using a modification of Bragg’s law:
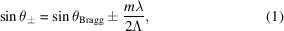
where 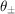 denotes the position of the satellite peak, 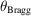 the position of the Bragg peak and 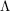 the bilayer period (Fallmann *et al.*, 2019 ▸; Clemens & Gay, 1987 ▸). The bilayer period derived from the spacing between satellites agrees with the nominal value of 20 nm within <5% error. Structural imperfections in the Cu/Mo superlattice, such as layer thickness fluctuations, interface roughness or crystalline disorder (Ariosa *et al.*, 2018 ▸; Moszner *et al.*, 2016 ▸), contribute to a noticeable broadening of the satellite peaks, which can interfere with accurate peak determination and residual stress evaluation at 0° tilt. Upon tilting the sample for stress analysis, the satellite peaks gradually disappear and only the main Bragg peak remains observable, which is then used for stress derivation.

**Figure 2:**
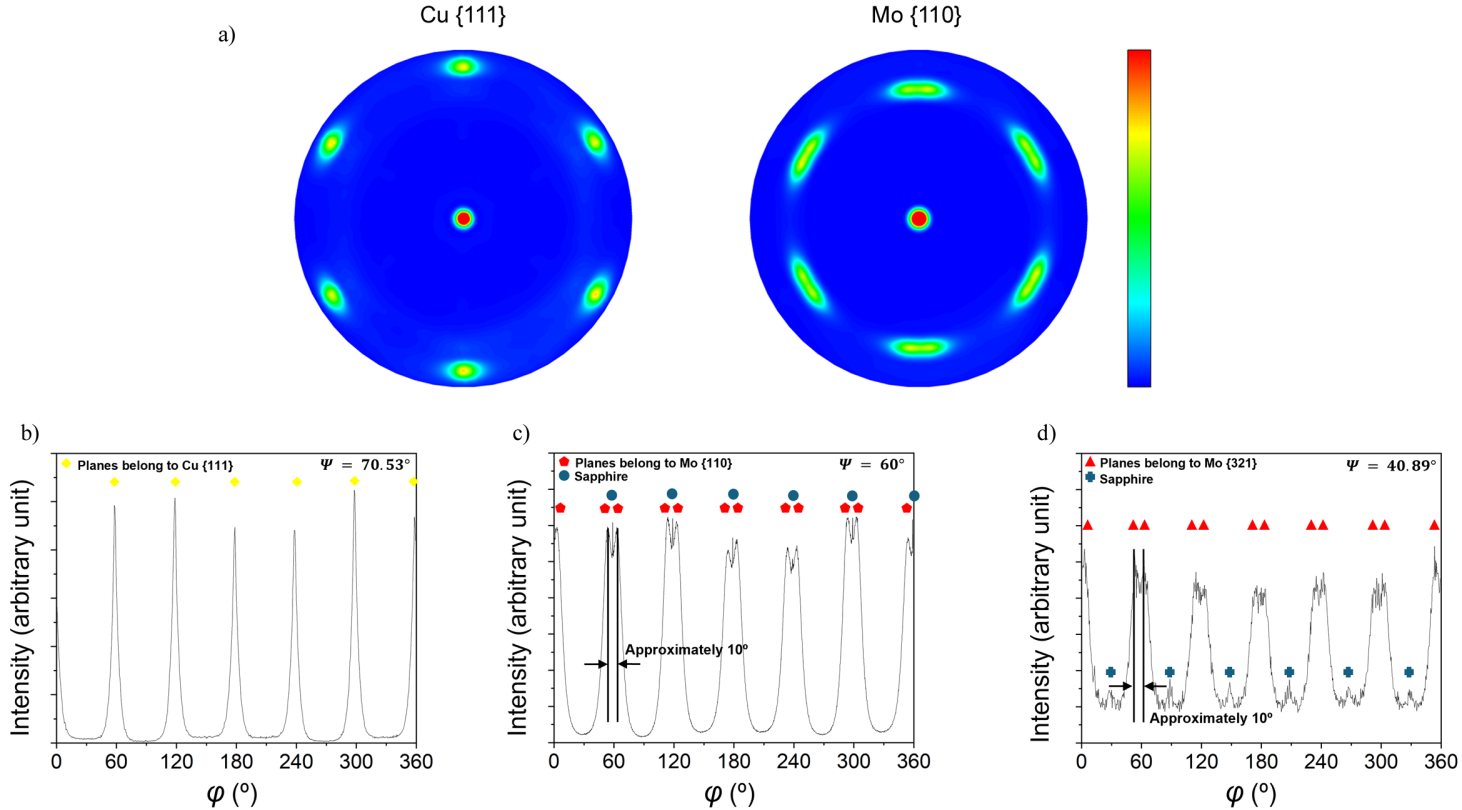
(*a*) XRD pole figures of the Cu {111} planes and the Mo {110} planes. (*b*) φ scan of the Cu {111} planes recorded at Ψ = 70.53°. (*c*) φ scan of the Mo {110} planes recorded at Ψ = 60°. (*d*) φ scan of the Mo {321} planes recorded at Ψ = 40.89°.

**Figure 3:**
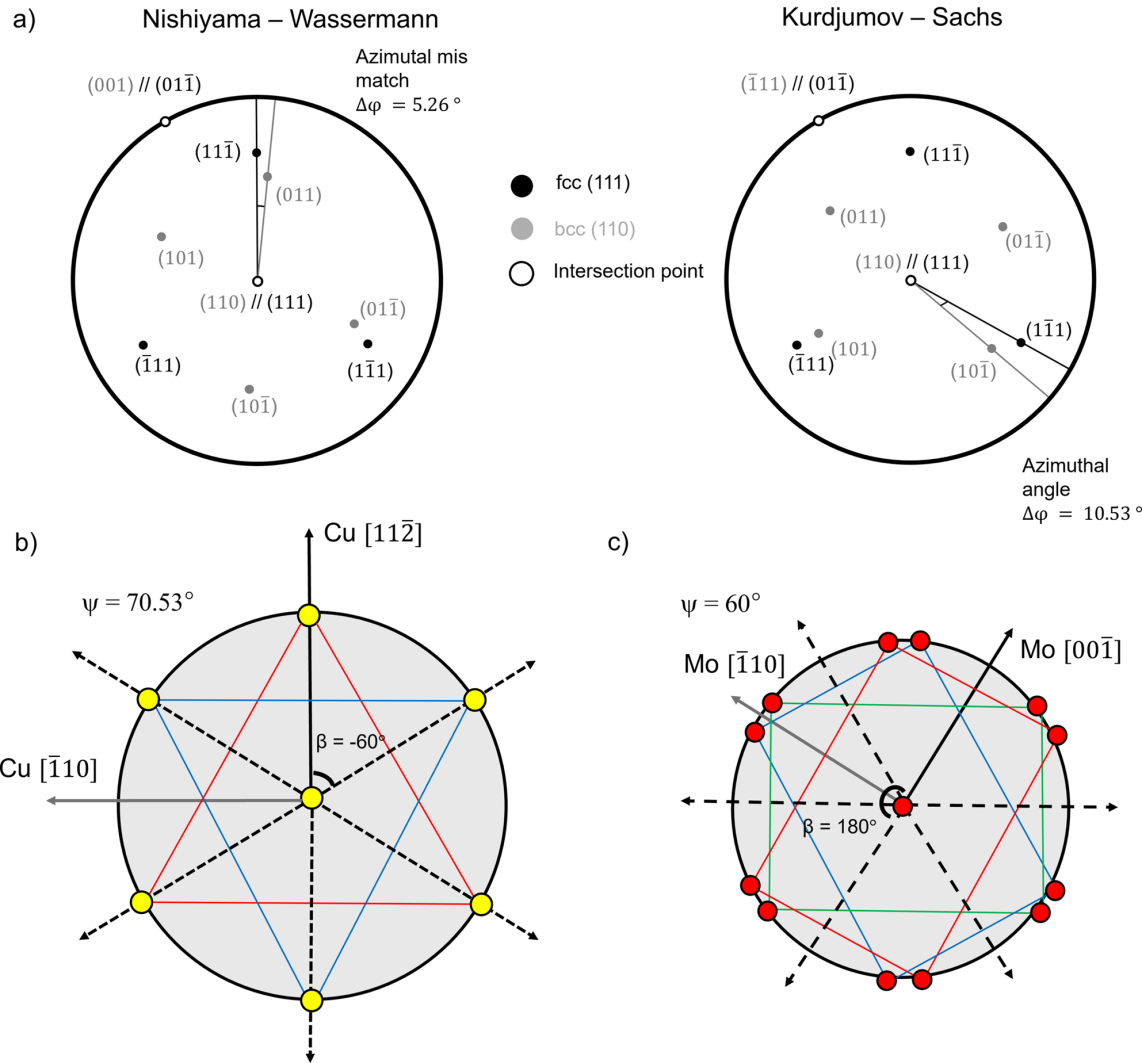
(*a*) Stereographic projection showing NW and KS crystallographic orientation (equivalent variants) for fcc (111) and bcc (110). (*b*) Illustration of the Cu {111} pole figure experiment in the presence of twinning. (*c*) Illustration of the Mo {110} pole figure experiment in the presence of twinning. The solid outer circle in panel (*b*) is at a tilt angle ψ = 70.53° and that in panel (*c*) at 60°; the solid black arrowed lines in panels (*b*) and (*c*) denoting Cu 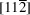 and Mo 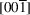, respectively, exhibit sixfold rotational symmetry when they overlap perfectly with the dashed lines. Here, β denotes a symmetrical angle (see Section 3.3 for a detailed description); in this context, it takes the values 60°, 120°, 180°, −60° and −120°. For example, panel (*b*) shows β = −60° and panel (*c*) shows β = 180°.

The θ–2θ scans indicate a pronounced out-of-plane texture, with the Cu {111} and Mo {110} planes oriented parallel to the sample surface, *i.e.* Cu (111)||Al_2_O_3_ (0001) and Mo (110)||Al_2_O_3_ (0001) ORs. The pole figures of the Cu {111} and the Mo {110} reflections, presented in Fig. 2 ▸(*a*), further reveal the presence of a well-defined in-plane texture, characterized by sixfold rotational symmetry. Intense poles appear at Ψ = 70.53° for Cu {111} and Ψ = 60° for Mo {110}. Notably, the Cu {111} pole figure is expected to exhibit threefold symmetry. The observed sixfold symmetry in Fig. 2 ▸(*a*) can be explained by the presence of growth twins in the Cu {111} layer, corresponding to a rotation angle of φ = 60° around the surface normal. This twinning leads to two equivalent in-plane orientations of the Cu lattice on the Al_2_O_3_ (0001) substrate. Similar quasi-epitaxial growth of Cu in two twinned variants on sapphire (0001) has been reported previously in the literature (Dehm *et al.*, 2005 ▸). Figs. 2 ▸(*b*)–2 ▸(*d*) show φ scans of the Cu {111} planes (Ψ = 70.53°), Mo {110} planes (Ψ = 60°) and Mo {321} planes (Ψ = 40.89°), respectively. These φ scans reveal that the high-intensity peaks possess nearly identical maximum intensities, indicating a relatively uniform distribution of crystallographic variants. Moreover, the φ positions of the sixfold Cu, Mo and sapphire peaks coincide, meaning that a specific in-plane OR exists between the layers and the substrate. The Mo {110} φ scan [Fig. 2 ▸(*c*)] shows a double-peak structure around the φ positions of the Cu {111} peaks [Fig. 2 ▸(*b*)], with a small central spike arising from the sapphire substrate. Similarly, the Mo {321} φ scan [Fig. 2 ▸(*d*)] shows substrate peaks alongside the main Mo reflections, highlighting the importance of carefully selecting diffraction planes within the accessible angular range to avoid substrate interference. The Mo {110} double peaks are separated by approximately 10° and each peak is shifted by approximately 5° relative to the φ positions of the Cu {111} planes [Figs. 2 ▸(*c*)–2 ▸(*d*). This shift originates from an NW OR between Cu and Mo lattices, *i.e.* Cu (111) 
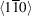
||Mo (110) 〈001〉, with an exact azimuthal mismatch of 5.26° between neighboring Cu {111} and Mo {110} peaks [Fig. 3 ▸(*a*)].

For bcc films grown on fcc layers, two ORs are most frequently observed: the Kurdjumov–Sachs (KS) and the NW. In the KS case, the relation is defined as fcc (111) 〈110〉||bcc (110) 〈111〉, while in the NW case it corresponds to fcc (111) 〈112〉||bcc (110) 〈001〉 (Fig. 3 ▸). Both ORs accommodate the atomic packing mismatch between close-packed planes of the fcc and bcc lattices, but they differ by a small relative in-plane rotation (∼5.26°). In this case, KS and NW ORs were distinguished experimentally by characteristic peak splitting, with sixfold rotational symmetry observed in the pole figures. Fig. 3 ▸(*a*) illustrates the NW and the KS ORs, while Figs. 3 ▸(*b*) and 3 ▸(*c*) display the Cu {111} and Mo {110} pole figure experiments, respectively [*cf*. Fig. 2 ▸(*a*)]. In the present case, the KS OR can be ruled out as it would require direct azimuthal coincidence between Cu 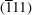 and Mo (101) reflections. In Fig. 3 ▸(*b*), the yellow circles denote the Cu {111} reflections, with the red and blue triangles representing the two twin variants of Cu. Fig. 3 ▸(*c*) shows the Mo {110} reflections, with red, blue and green rectangles representing the three Mo {110} variants according to the NW OR. For the present system, the ORs for the two Cu variants with respect to the sapphire substrate and the Mo layer can be expressed as Cu 
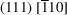
||Al_2_O_3_ 
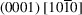
||Mo 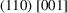 and Cu 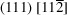
||Al_2_O_3_ 
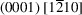
||Mo 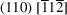. Importantly, both the Cu (111)/sapphire OR, established by the initial Cu layer, and the NW OR between Cu (111) and Mo (110), are maintained throughout the Cu/Mo NML stack, as confirmed by the corresponding poles in the pole figures of Fig. 2 ▸.

### 3.2. Microstructural analysis

**Figure 4:**
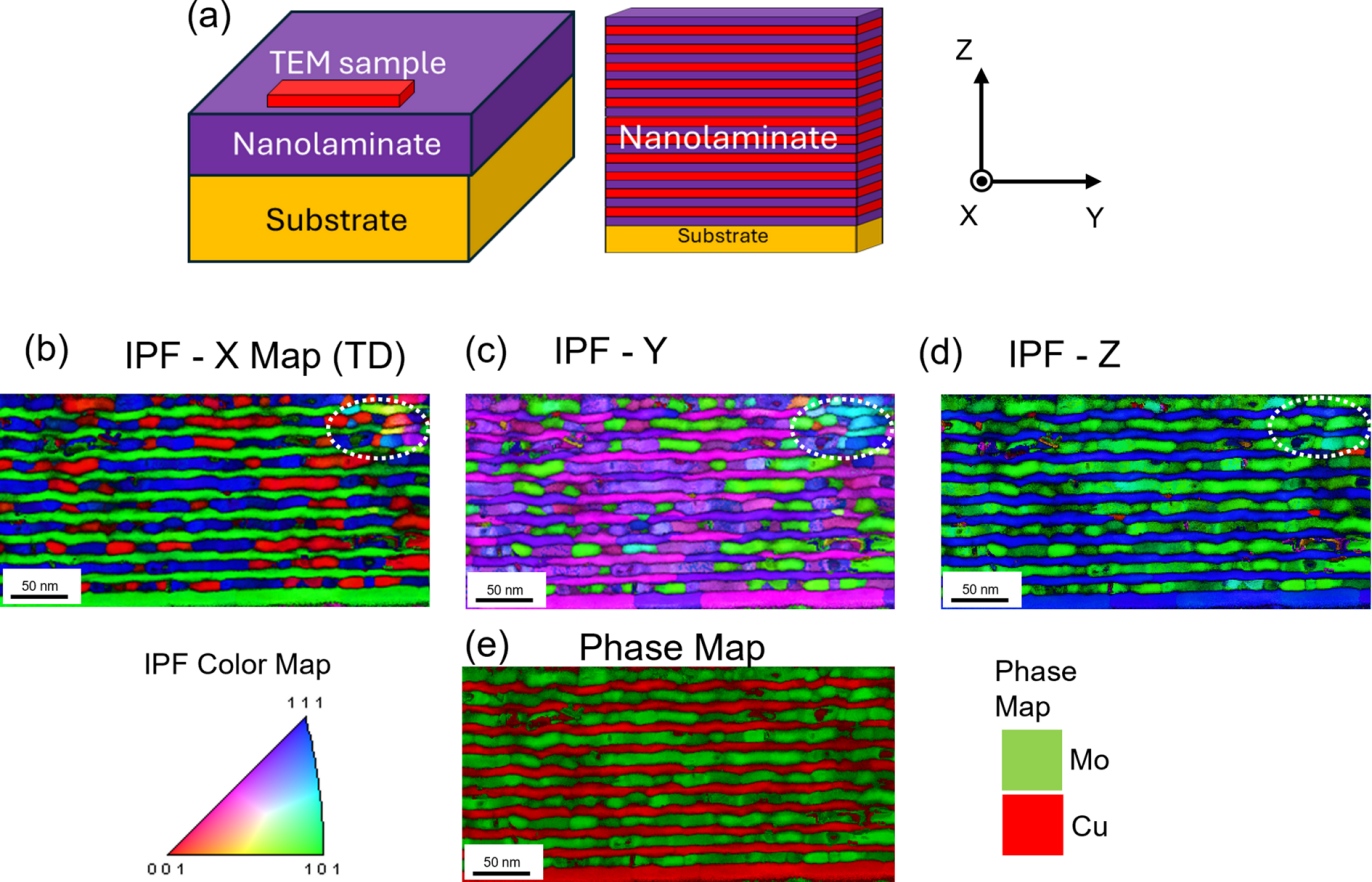
(*a*) The sample and its geometric relationship to the TEM lamellae (note that the sample is rotated by 90° with respect to the pole figure orientations in Fig. 2). (*b*)–(*d*) SPED-derived IPF maps for the *X*, *Y* and *Z* directions. (*e*) Phase map.

The microstructure of the NMLs, including surface and cross-sectional aspects, was characterized by SEM and STEM analysis, with additional detailed information provided in the supporting information. Elemental distribution and crystallographic phase mapping to derive the in-plane OR have been carried out. Fig. 4 ▸(*a*) illustrates the geometric relationship of the TEM lamella with respect to the NML sample and presents a cross section taken in the *YZ* plane. Figs. 4 ▸(*b*)–4 ▸(*d*) show the inverse pole figure (IPF) maps for the *X*, *Y* and *Z* directions, respectively, with the sample directions defined in Fig. 4 ▸(*a*). Fig. 4 ▸(*e*) displays the phase map. Based on the φ scan and pole figure results, Figs. 4 ▸(*b*)–4 ▸(*d*) clearly show the distribution of the NW growth variants, in agreement with the XRD results. For instance, in Fig. 4 ▸(*b*) Mo is visible in two distinct orientations of 〈001〉 (NW OR variant 1) and close to 〈111〉 (NW OR variants 2 and 3; the slight deviation of 5.26° is not discernible). Fig. 4 ▸(*d*), with crystallographic information along *Z*, *i.e.* along the growth direction, again displays the out-of-plane texture of Cu (111)||Mo (110). Additionally, a localized area with non-NW ORs is observed, marked by the white dotted ovals in Figs. 4 ▸(*b*)–4 ▸(*d*).

### 3.3. Transformation of coordinates for stress analysis

Accurate evaluation of residual stresses in strongly textured NMLs requires careful consideration of the crystallographic texture, which is achieved through the use of appropriate coordinate systems. The diffraction signal is measured in the laboratory coordinate system (L), whereas the elastic strain depends on the crystal orientation and the stress tensor must ultimately be expressed in the sample coordinate system (P) for macroscopic interpretation. Consequently, defining a consistent mathematical framework of coordinate transformations between these systems is essential. In the following, we present the transformation relations linking laboratory, crystal and sample coordinates, for the specific case of Cu {111} and Mo {110} out-of-plane textures, which form the basis for residual stress determination in the Cu/Mo NML.

The crystallographic coordinate system X, with axes X_*i*_ (*i* = 1, 2, 3) originally aligned along the [100], [010] and [001] directions of the cubic crystal, is introduced as the reference frame in which the elastic constants are defined. For a given texture component or grain orientation, this system is rotated into an orientation-dependent crystal coordinate system 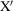, whose axes are defined by the crystallographic directions corresponding to the considered orientation. The explicit form of 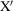 is specified once the texture and diffraction geometry are introduced. (Note that these X and X′ systems are specific to the crystal’s orientation and are distinct from any coordinate systems used in Fig. 4 ▸ for TEM purposes.)

**Figure 5:**
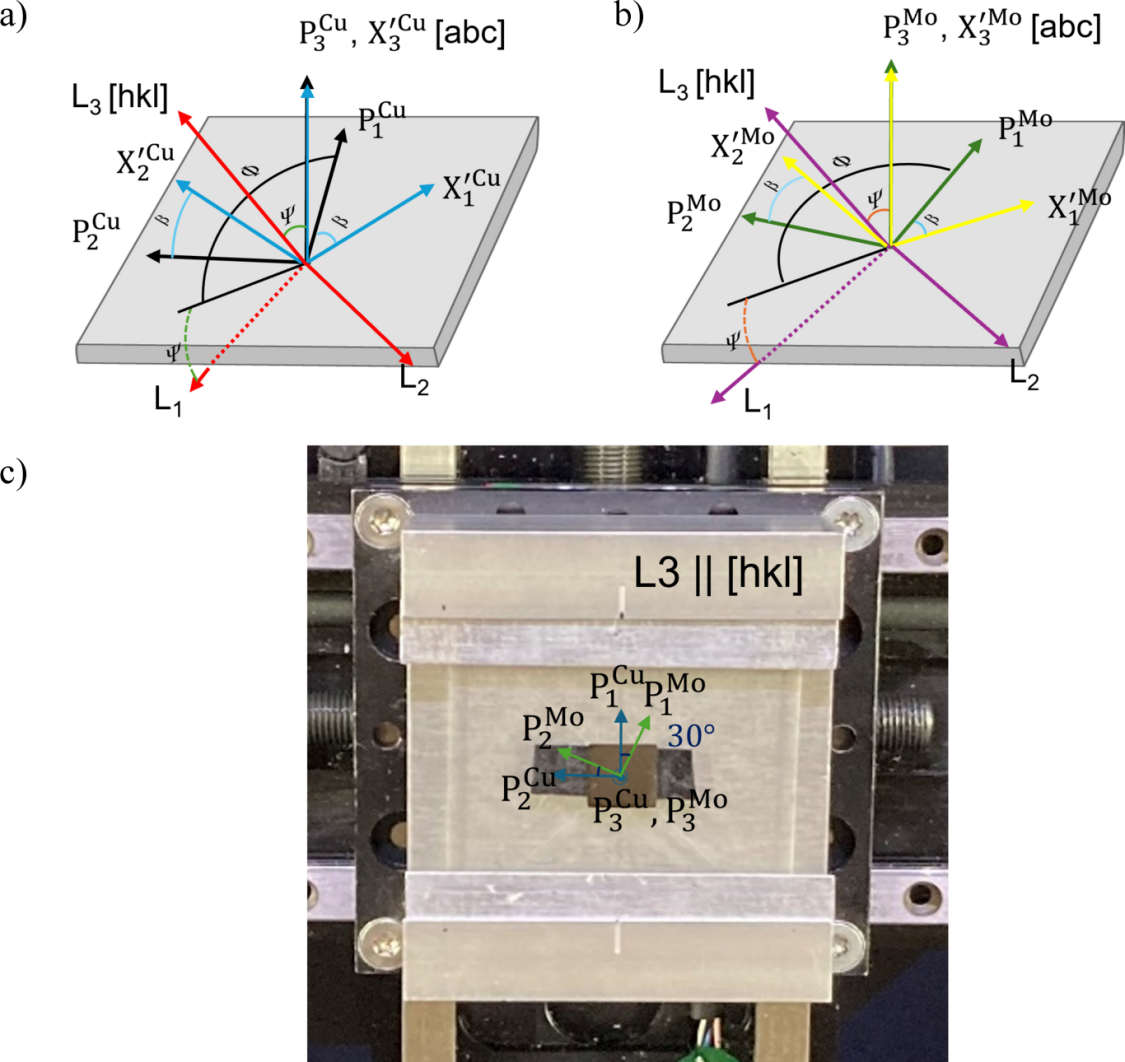
Specimen coordinate system P, orientation-dependent crystallographic coordinate system 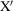 and laboratory coordinate symtem L for (*a*) Cu and (*b*) Mo. (*c*) Plane view of an experimental sample with coordinates of specimen P for Cu and Mo when β = 0° (the 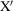 and P axes are aligned). Sharing a common 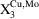 axis (sample normal), the Mo crystallographic coordinate system is oriented 30° clockwise relative to the Cu system about this axis. For the Mo layer, the coordinate system P^Mo^ is defined following convention and to remain consistent with the reference, such that stresses are evaluated in a frame rotated with respect to P^Cu^ according to the crystallographic OR between Cu and Mo. In this framework, the stress tensor of each layer is determined with respect to its corresponding coordinate system. Although referred to as a specimen coordinate system by convention, P^Mo^ should be understood as a layer-specific reference frame used for stress evaluation.

The reference frames of the specimen (P), orientation-dependent crystal (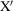) and laboratory (L) coordinate systems are illustrated in Figs. 5 ▸(*a*)–5 ▸(*c*). In this study, Cu {111} and Mo {110} out-of-plane textures were observed; thus, 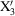 is defined to coincide with Cu [111] or Mo [110], respectively. The 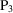 axis is likewise chosen to align with the 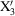 axis. The 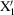 and 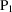 axes are related by a rotation of an angle β about the common 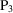 (or 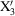) axis. The specimen and laboratory coordinates are related by the rotational angles Φ and Ψ about the P_3_ and L_2_ axes, respectively [see Figs. 5 ▸(*a*)–5 ▸(*c*)]. Note that the rotational angle Φ is defined as the angle between the P_1_ axis and the line formed by the projection of L_1_ onto the sample surface.

NMLs exhibiting pronounced in-plane twin variants pose a challenge for accurate residual stress analysis, as their complex crystallographic texture deviates significantly from that of an ideal single crystal. To address this issue, an additional degree of freedom is introduced into the stress–strain relationship through the angle β. This angle explicitly defines the orientation of the crystal axes relative to the specimen coordinate system, thereby capturing the range of in-plane rotations present among individual crystallites. Specifically, β represents the collective orientation of crystallites whose rotational symmetry about the sample normal satisfies the Bragg condition and thus contributes to the measured intensity. Conceptually, by systematically varying β within a single-crystal framework, the overall texture of the sample can be effectively reconstructed. This approach allows us to account for the collective contribution of symmetry-related crystallites to the residual stress in a coherent manner, employing a Reuss-type averaging over the contributing crystallite variants. Note that β is not an independent experimental angle but is intrinsically coupled to the specimen rotation angle Φ. Consequently, diffraction from a given (*hkl*) plane occurs only for discrete combinations of (Φ, β), which are determined by the material’s OR and texture symmetry. By explicitly incorporating β into the coordinate transformation, the modified method developed in this work substantially extends the applicability of the CGM. This allows for accurate residual stress determination in epitaxial multilayers exhibiting strong in-plane texture and twinning.

**Figure 6:**
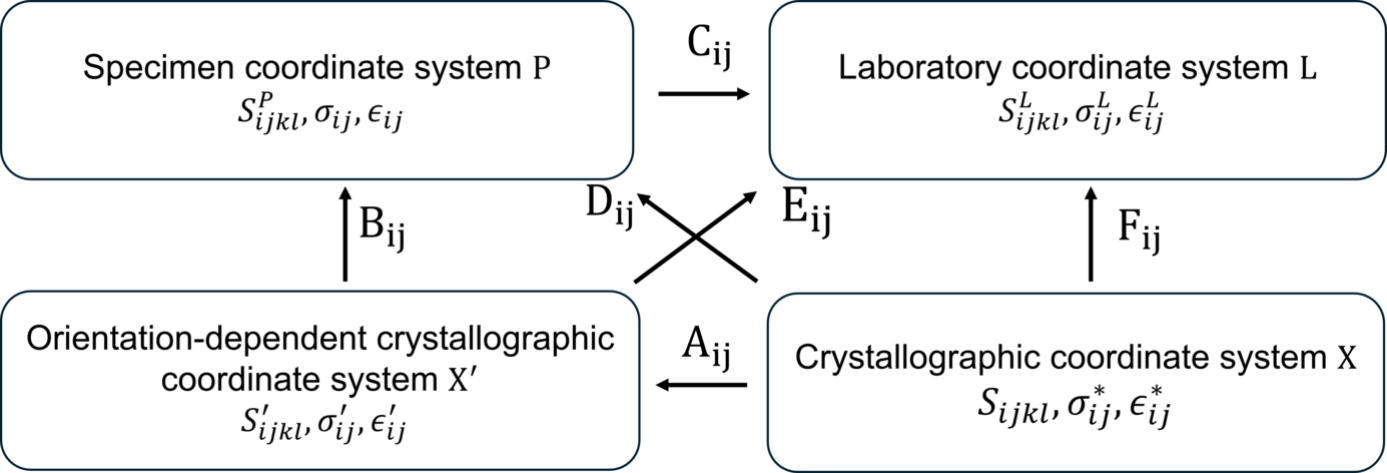
Illustration of the transformation relationship between different coordinate systems. *A_ij_*, *B_ij_*, *C_ij_*, *D_ij_*, *E_ij_* and *F_ij_* denote the components of the respective transformation matrices, 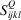 represents elastic compliance in *Q* coordinates, and 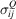 and 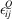 represent stress and strain, respectively, in *Q* coordinates.

The rotational transformation of vector components between coordinate systems can be represented by an orthogonal matrix as follows:
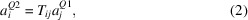
where 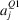 and 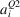 represent the vector components in the original (*Q*1) and transformed (*Q*2) coordinate systems, respectively, and 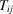 denote the components of an orthogonal transformation matrix. The transformation of stress (or strain) and elastic compliance (or stiffness) matrix components can be obtained by equations (3) and (4), respectively:
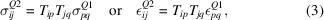

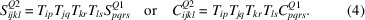
Here, 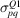 and 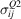 (or 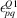 and 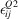) denote stress (or strain), while 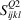 and 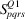 (or 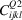 and 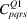) denote elastic compliance (or stiffness). The relationship between the different coordinate systems is depicted in Fig. 6 ▸.

Following the above definition of coordinate systems for Cu with (111) and Mo with (110) out-of-plane textures, the components of the transformation matrices *A_ij_* between crystallographic and crystal coordinate systems can be expressed as
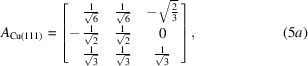

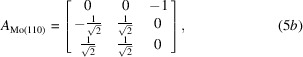
where 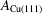 is the matrix for Cu, with axes 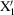 = 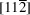, 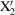 = 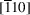 and 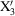 = [111], and 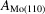 is the matrix for Mo with axes 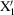 = 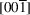, 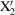 = 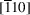 and 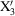 = [110].

The transformation matrix components 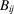 and 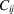, which hold for both crystallographic phases, can be expressed as
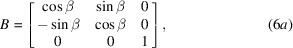

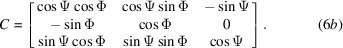


#### 3.3.1. {111} out-of-plane texture: the case of Cu

We evaluate the out-of-plane strain component in the laboratory frame, 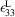, for residual stress determination by XRD. The relations used are as follows:
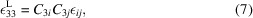

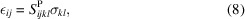

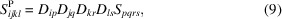

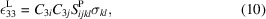

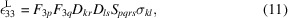
with
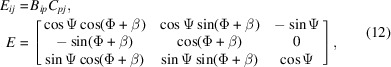

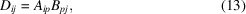

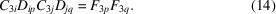
In equation (13), the index Cu (111) for 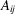 has been dropped for the sake of simplicity.

Residual stress in a material manifests as shifts in the diffraction peak positions, which arise from elastic strains in the crystallites. In the case of a perfect single crystal, all diffracting domains share the same orientation and the measured lattice strain directly reflects the stress state associated with that orientation. In real materials, however, even when a pronounced crystallographic texture is present, a certain degree of polycrystallinity always exists. Consequently, the measured diffraction signal represents an average response over many differently oriented grains. To relate the measured lattice strains to the macroscopic stress state, a grain interaction model is required. Within the Reuss model, it is assumed that all crystallites in the polycrystal experience the same macroscopic stress, while their elastic strains vary according to their crystallographic orientation and elastic anisotropy.

On the basis of this assumption, an expression linking the average stress tensor components in the specimen coordinate system 〈σ_*ij*_〉 to the macroscopic stress tensor components σ_*ij*_ can be written as
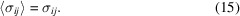


In the present work, the Reuss model is considered and the bracket notation is omitted for simplicity. Microscopic strain variations are not considered in the present analysis; the evaluation is restricted to macroscopic residual stresses derived from diffraction peak positions. While local stress components such as σ_3*j*_ may in principle exist at the microscopic level or within individual orientation variants, XRD measures an averaged stress state over the irradiated volume. For the thin-film geometry considered here, a macroscopic biaxial in-plane stress state is therefore assumed, such that
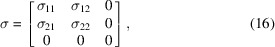
where 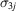 ≃ 0 at the macroscopic level.

By combining this assumption with the above transformation relations, the strain in the laboratory coordinate system 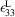, with crystal axes defined as 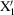 = 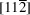, 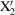 = 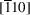 and 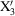 = [111], can be expressed as (Tanaka *et al.*, 1996 ▸, 1999 ▸)
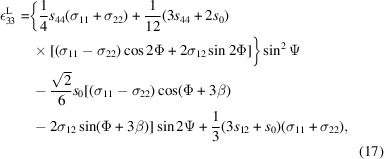
where 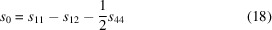
is the anisotropy term and *s*_11_, *s*_12_ and *s*_44_ are the components of the elastic compliance tensor of a cubic crystal expressed in Voigt notation.

**Figure 7:**
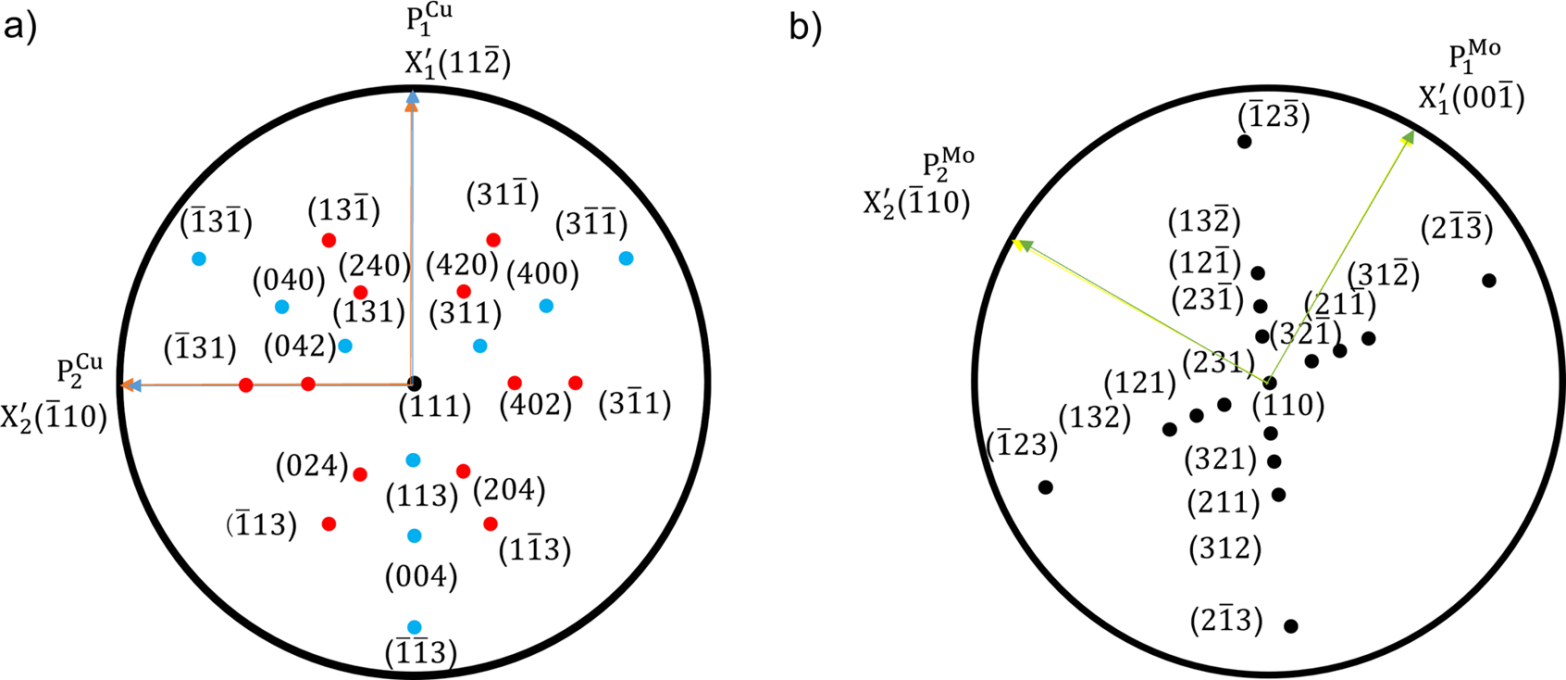
Schematic representations of crystallographic plane symmetries relevant to XRD reflections in cubic crystals, illustrated using stereographic projections: (*a*) [111]-oriented and (*b*) [110]-oriented configurations. Planes exhibiting threefold and sixfold rotational symmetry are highlighted in blue and red, respectively.

The diffraction planes were selected to minimize overlap with substrate reflections and superlattice satellite peaks, to ensure clear separation between Cu and Mo reflections and to preferentially employ higher 2θ angles, thereby improving the accuracy of peak position determination in the residual stress analysis. The selected diffraction planes are summarized in Table 1 ▸, which also distinguishes for the case of Cu between those exhibiting a threefold and sixfold rotational symmetry [Table 1 ▸ and Fig. 7 ▸(*a*)]. Note that the threefold symmetry refers to the intrinsic crystallographic symmetry of a single fcc (111) orientation when described in stereographic projection, whereas the sixfold symmetry corresponds to an effective symmetry emerging from the superposition of multiple orientation variants.

Let us consider first the case of reflections exhibiting sixfold rotational symmetry, as exemplified by the representative reflection planes of (420) at Ψ = 39.23°. Starting from the (204) plane (as an example), the other symmetry-related planes are (240), (420), (042), (402), (024) and (204) (Table 1 ▸ and Fig. 7 ▸). To measure the (204) peak specifically, Φ should be set to −150° with Ψ = 39.23°. Note that Φ is a goniometer-controlled variable associated with the laboratory frame, whereas β is a conceptual angle that rotates the chosen single-crystal frame to reproduce the sample’s texture. Unlike the single-crystal assumption in conventional CGM, β is used here to quantify how the actual texture influences residual stress. So, while Φ is the rotation one applies experimentally with the goniometer, β is an intrinsic orientation offset that distinguishes between the different orientation variants allowed by the epitaxial relationship and twinning. Owing to the sixfold rotational symmetry in the pole figure [Figs. 5 ▸(*a*) and 5 ▸(*b*)], the measured peak can be regarded as receiving equal contributions from the (240), (420), (042), (402), (024) and (204) planes. In practice, β is varied on the chosen single-crystal frame in 60° increments to account for the symmetry-related orientations. Averaging over β is therefore justified, as the φ-scan intensities of these planes are similar [see Figs. 2 ▸(*b*)–2 ▸(*d*)]. Consequently, equation (17), which assumes a single crystal, cannot be applied directly; β must be interpreted as an angle used to determine an average value related to the texture. Importantly, β is not an independent variable but depends on the angle Φ. For a given (*hkl*) plane, when Φ and Ψ are set to excite diffraction, β for that (*hkl*) reflection is 0 and serves as the reference. The contributions of symmetry-related planes are then incorporated conceptually by varying β and averaging. For practical use with Table 1 ▸, β can be referenced to Φ = 0°; for any general Φ, β in equation (17) should be replaced by β − Φ. Equation (17) then reads
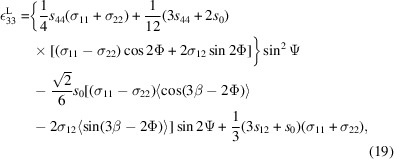
where angle brackets 〈 〉 represent an average value. For this reason, equation (19) should be averaged with β = 30°, −30°, 90°, −90°, 150° and −150°. The averaging of 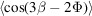 and 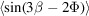 yields 
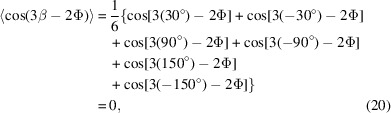
and
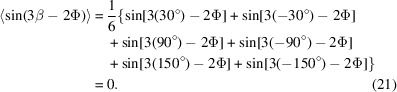


For Cu diffraction planes with threefold rotational symmetry, such as the representative plane reflection of (311), the corresponding symmetry-related planes are (131), (311) and (113) [Table 1 ▸ and Fig. 7 ▸(*a*)]. The (311) peak can be obtained when Φ is set to −60° at Ψ = 29.5°, as shown in Table 1 ▸. The measured peak can be regarded as coming equally from the (131), (311) and (113) planes, which – consistent with the reasoning given above – implies averaging β values of 60°, −60° and 180°. In this case, the averaging of 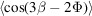 and 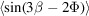 yields
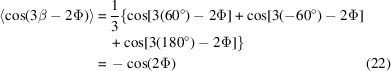
and
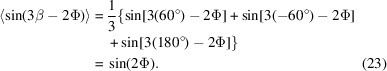


For diffraction planes exhibiting threefold rotational symmetry, the modified approach is consistent with the conventional CGM. This can be verified directly without averaging using the allowed β values. For example, when β = −60°, 60° or 180°, the values of 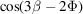 and 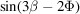 match equations (22) and (23), confirming that both methods yield equivalent residual stress expressions. In contrast, for sixfold rotational symmetry, this is no longer the case. For example, when β = −150° in Table 1 ▸ (corresponding to Φ = −150° in the CGM convention), 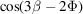 and 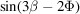 do not coincide with equations (20) and (21), highlighting the deviation from the standard CGM formulation.

#### 3.3.2. (110) out-of-plane texture: the case of Mo

Using a similar rationale to that described in Section 3.3.1 and under the same assumptions of in-plane biaxial stress and Reuss model, the strain in the laboratory coordinate system 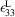, with the crystal axes defined as 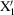 = 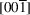, 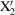 = 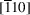 and 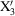 = [110], can be expressed as (Tanaka *et al.*, 1999 ▸, 2000 ▸)
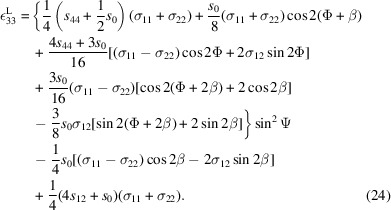


To utilize the orientation angle in Table 2 ▸ for Φ = 0°, one must replace β in equation (24) with β − Φ, which after averaging over β leads to
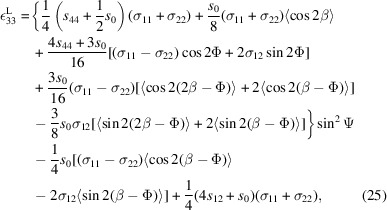
where again angle brackets represent an average value.

Using the same criteria for selecting diffraction planes, the corresponding planes and their orientation angles are summarized in Table 2 ▸ [see also Fig. 7 ▸(*b*)]. For the Mo (110) textured layer, the diffraction planes have β values of 35.26°, −35.26°, 144.74° and −144.74°. The averaging procedure, however, differs from that used for the {111} out-of-plane texture. Consider, for instance, the representative plane reflection of (123) at Ψ = 40.89°. As depicted in Figs. 2 ▸(*a*), 2(*c*) and 2(*d*), Mo exhibits sixfold rotational symmetry with comparable maximal intensities. The measured intensity only originates from 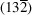 and (312), because only those planes overlap with the sixfold rotationally symmetric points observed experimentally. As seen in Fig. 7 ▸(*b*), other planes like 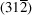 and (132) do not coincide with this symmetry and therefore do not contribute to the peak intensity. Consequently, non-overlapping planes are excluded from average calculations. In this case, Φ = −144.74° and β = 35.26° and −144.74° should be chosen, and thereby the averaging process is
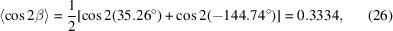

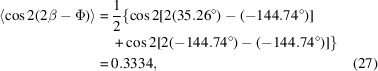

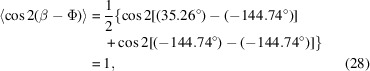

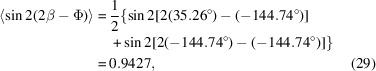

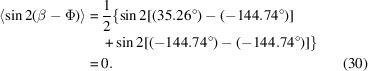


#### 3.3.3. Evaluation of residual stress

In this work, the stress analysis is performed using a least-squares fitting procedure applied to the experimentally measured lattice strains, taking into account the elastic anisotropy of the constituent layers and the accessible diffraction geometries. This approach allows reliable determination of the in-plane residual stress components, while also providing a robust framework to evaluate the influence of texture and data quality on the extracted stress values. The 2θ peak positions were determined by fitting the diffraction profiles with a pseudo-Voigt function, from which the strained lattice parameters were calculated using Bragg’s law. The in-plane residual stress components, σ_11_, σ_22_ and σ_12_, in the Mo (110) and Cu (111) sublayers were then determined by fitting equations (19) and (25) using a nonlinear least-squares algorithm implemented in MATLAB. Details of the fitting procedure, including the formulation of the weighted residual function and the derivation of parameter uncertainties, are provided in the supporting information. The corresponding sin^2^Ψ fit curves and residual plots for both Mo and Cu datasets are also shown in the supporting information to allow full evaluation of the quality and robustness of the stress determination. The analytical expressions for Cu and Mo and the specific diffraction planes used for the stress evaluation are likewise provided in the supporting information. The elastic compliances employed in the calculations were taken from values of single-crystal Cu and Mo, as listed in Table 3 ▸.

For Cu, diffraction lines with both three- and sixfold rotational symmetry were selected for the evaluation of residual stress. The resulting values of residual stress for both Cu and Mo sublayers are listed in Table 4 ▸.

The residual stresses in Cu were evaluated separately from diffraction peaks exhibiting threefold (Cu_1_) and sixfold (Cu_2_) rotational symmetry in order to distinguish diffraction groups governed by different analytical strain–stress relations. This methodological distinction enables a consistent treatment of symmetry-related diffraction conditions within the framework of the present analysis. The resulting stress values differ by approximately a factor of two. This difference does not indicate a distinct physical stress state but reflects the different statistical constraints of the two analyses: Cu_2_ is based on only two diffraction points, resulting in a less constrained fit and larger uncertainty (>0.25 GPa) whereas Cu_1_ is supported by three diffraction points per Φ angle, yielding a more reliable estimation. For this reason, we have not reported formal confidence intervals for Cu_2_ in Table 4 ▸. With only two data points entering the fit, the statistical degrees of freedom are extremely limited and a meaningful error estimation from residual-based covariance analysis would not be robust. The Cu_2_ values are therefore provided as supplementary methodological results illustrating the influence of diffraction symmetry selection, while Cu_1_, with its larger data basis and well-defined uncertainties, is used for quantitative comparison with the Mo stresses. Nevertheless, the two analyses exhibit similar trends in the stress components. The stress-free lattice parameters were also fitted for Mo and Cu; they are within 0.002 Å of the bulk values for Mo and 0.004 Å for Cu, indicating a minor strain offset and confirming the accuracy of the stress–strain determination procedure.

The residual stress state in both Cu and Mo layers can be considered nearly equi-biaxial, since the shear component σ_12_ has an insignificant magnitude compared with σ_11_ and σ_22_. We emphasize that the residual stress state in the Cu/Mo multilayers shows an opposite sign in the two constituent layers: the Cu layers are under tensile stress, while the Mo layers are under compressive stress. This behavior is consistent with the large lattice mismatch between fcc Cu (111) and bcc Mo (110) planes when adopting the NW OR: −18.8% along Cu 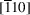
||Mo [001] and −0.5% along Cu 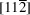
||Mo 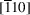. In this OR, the Cu {111} planes are parallel to Mo {110}, but the in-plane atomic spacings do not match perfectly, leading to coherency stresses at the interfaces. To accommodate this mismatch, the Cu layers tend to expand laterally, giving rise to tensile stress, whereas the Mo layers are constrained and thus develop compressive stress of similar magnitude. For quantitative comparison with Mo, the Cu_1_ stress value together with its standard deviation is taken as the representative Cu stress. Within experimental uncertainty, the Cu and Mo stresses are comparable in order and opposite in sign. This confirms that the dominant contribution arises from coherency stresses imposed by the NW OR, which are redistributed between the two constituents in order to maintain mechanical equilibrium of the multilayer stack. Because multiple symmetry-equivalent in-plane variants (*e.g.* twinning and rotational variants) form under the NW OR and are locked into well-defined epitaxial relationships with the sapphire substrate and with each other, the in-plane directions are, on average, symmetry equivalent when probed by XRD. As a result, any anisotropic local stress variations between crystallographic variants are effectively averaged out, yielding macroscopic in-plane stresses that are nearly identical. The observation of an equi-biaxial stress state therefore reflects both the crystallographic symmetry of the NW OR and the relaxation mechanisms (*e.g.* twinning, misfit dislocations) that help distribute the coherency stress iso­tropically within the film plane.

### 3.4. HR-STEM

**Figure 8:**
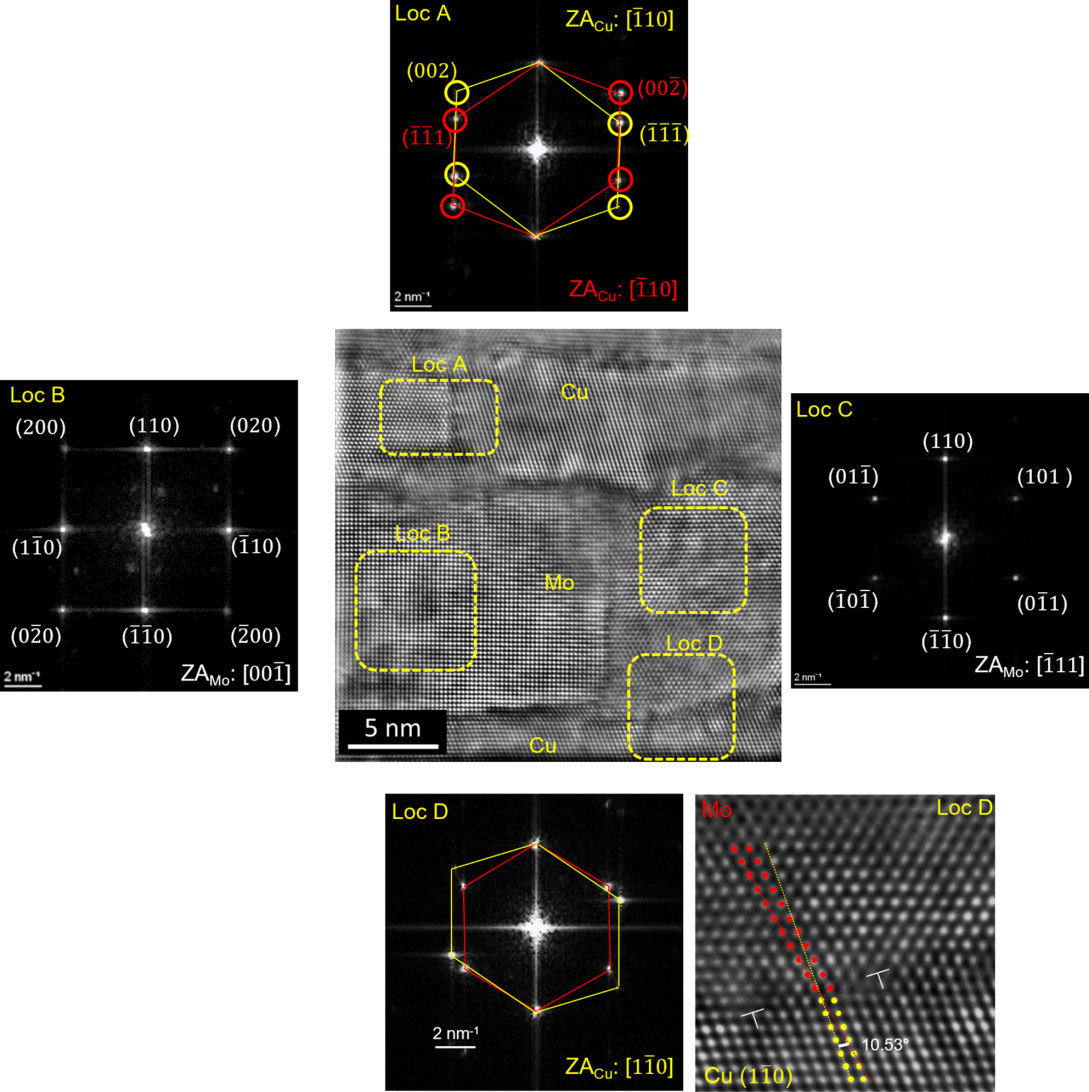
(Center) HR HAADF STEM image of Cu/Mo NMLs. Around this are FFTs of selected regions of the central image and a locally magnified image obtained from Loc D in the central image.

In the present study, HR-STEM was employed to complement the diffraction-based stress analysis by visualizing the layer morphology and verifying the crystallographic ORs between Cu and Mo. This technique also allows the identification of misfit dislocations and interfacial defects, which are crucial for understanding stress relaxation mechanisms and the overall microstructural stability of the multilayers. As revealed in Fig. 8 ▸ [comprising an HR HADDF STEM image, the fast Fourier transforms (FFTs) of selected regions and a magnified view], the interface exhibits a semi-coherent structure with a local texture of Cu (111)||Mo (110) out of plane. In Fig. 8 ▸, Loc A shows two Cu grains with zone axes (ZA) 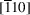 in a twin relation, in agreement with the XRD pole figures shown in Section 3.1. Loc B and Loc C show Mo grains with 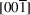 ZA and 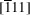 ZA, respectively (a misalignment of 5.26° is expected with regard to the XRD results reported in Section 3.1). In Loc D, the 10.53° angle between Cu (111) and Mo (011) is the expected angle between the out-of-plane texture of Cu (111) and Mo (110) parallel to each other. In Fig. 2 ▸, the tilt angle difference between the (111) Cu and (110) Mo planes is indeed 70.53° − 60° = 10.53°.

The presence of misfit dislocations at this interface can partially relieve the in-plane lattice mismatch between Cu and Mo (Politi *et al.*, 2000 ▸) exhibiting the NW OR. Another important contributing factor is the effective sixfold rotational symmetry that was observed in the pole figures and directly caused by growth twinning. Unlike the potentially severe residual stress expected in a perfectly NW-oriented crystal due to coherency stress, this symmetry, in conjunction with observed defects and localized regions exhibiting non-NW ORs [as shown in Figs. 4 ▸(*b*)–4 ▸(*d*)], collectively promotes the effective relief of residual stress.

In previous studies (Yeom *et al.*, 2024 ▸, 2023 ▸) of fiber-textured Cu/Mo NMLs, which employed an Si substrate, both Cu and Mo layers exhibited compressive stress under the same deposition conditions. However, our measurements reveal residual stress values that are approximately equal and opposite in the two constituent sublayers, with similar magnitudes. Given that coherency stresses are inherently substantial, and equal and opposite, in the two constituents (Bain *et al.*, 1972 ▸), our findings therefore strongly suggest that the in-plane texture plays a critical role in mediating this dominant stress component, thus profoundly influencing the overall residual stress state.

## 4. Summary and conclusions

The theoretical framework and methodology for selecting diffraction planes to evaluate residual stresses in fcc and bcc nanomultilayer (NML) structures – characterized by strong in-plane and out-of-plane textures and the presence of twinning – have been established using XRD. The texture and residual stresses were experimentally investigated for representative NMLs composed of immiscible materials with an fcc/bcc structure, namely Cu/Mo multilayers grown on a sapphire (0001) substrate. The system exhibited a Nishiyama–Wassermann orientation relationship, with twinning resulting from the mutual adaptation of fcc Cu (111) planes and bcc Mo (110) planes, leading to a sixfold rotational symmetry in the pole figures.

We introduced β (redefined for the texture) as a growth direction rotation parameter to accurately capture the growth direction rotational symmetry, such as that arising from twinning. Owing to the similar peak intensities observed in the φ scans, averaging over β was justified to account for equivalent diffraction plane contributions. The residual stress in Cu, evaluated using diffraction points with both threefold and sixfold rotational symmetry, differed by approximately a factor of two, although the values were similar in magnitude and trend. This indicates that both diffraction plane selection strategies yield consistent results, validating the robustness of the proposed methodology.

Although the framework was applied here to the NW OR with twinning, the approach and discussion are broadly applicable to other orientation relationships in textured multilayer systems.

## 5. Related literature

For further literature related to the supporting information, see Druzhinin *et al.* (2019 ▸), Monclús *et al.* (2014 ▸), Moszner *et al.* (2016 ▸) and Srinivasan *et al.* (2006 ▸).

## Supplementary Material

Additional images and detailed derivations. DOI: 10.1107/S1600576726003638/xx5096sup1.pdf

## Figures and Tables

**Table 1:** Diffraction planes (representative) and orientation angles (Ψ and β at Φ = 0°) for the [111] out-of-plane texture The (*hkl*) listed under ‘Representative plane’ denote representative indices; for each β value, the specific symmetry-equivalent plane within the {*hkl*} family is indicated in parentheses.

	Representative plane				
	(311)	(311)	(400)	(311)	(420)
Tilt angle Ψ (°)	29.5	79.98	54.74	58.52	39.23
β (°) with Φ = 0°	60° (131)	60° ( 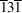 )	60° (040)	30° ( 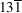 )	30° (240)
	−60° (311)	−60° ( 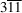 )	−60° (400)	−30° ( 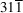 )	−30° (420)
	180° (113)	180° ( 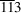 )	180° (004)	90° ( 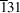 )	90° (042)
				−90° ( 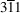 )	−90° (402)
				150° ( 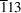 )	150° (024)
				−150° ( 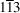 )	−150° (204)

**Table 2:** Diffraction planes (representative) and orientation angles (Ψ and β at Φ = 0°) for the [110] out-of-plane texture The (*hkl*) listed under ‘Representative plane’ denote representative indices; for each β value, the specific symmetry-equivalent plane within the {*hkl*} family is indicated in parentheses.

	Representative plane			
	(123)	(123)	(123)	(211)
Tilt angle Ψ (°)	19.11	40.89	79.11	30
β (°) with Φ = 0°	35.26° ( 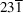 )	35.26° ( 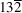 )	35.26° ( 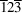 )	35.26° ( 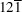 )
	−35.26° ( 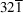 )	−35.26° ( 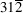 )	−35.26° ( 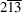 )	−35.26° ( 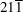 )
	144.74° (231)	144.74° (132)	144.74° ( 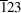 )	144.74° (121)
	−144.74° (321)	−144.74° (312)	−144.74° ( 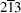 )	−144.74° (211)

**Table 3:** Compliance of single-crystal Cu and Mo

	*s*_11_	*s*_12_	*s*_44_	Reference
Cu	14.963 TPa^−1^	−6.268 TPa^−1^	13.266 TPa^−1^	Chang & Himmel (1966 ▸)
Mo	2.622 TPa^−1^	−0.690 TPa^−1^	9.363 TPa^−1^	Bolef & Klerk (1962 ▸)

**Table 4:** Residual stresses in Cu and Mo sublayers obtained from the modified CGM analysis Cu_1_ and Cu_2_ indicate the residual stresses evaluated by using diffraction planes with threefold and sixfold rotational symmetry, respectively. Note that the crystal axes for residual stress analysis of Cu and Mo sublayers are different. [See Fig. 5 ▸(*c*).]

	σ_11_	σ_22_	σ_12_
Cu_1_	0.22 ± 0.1 GPa	0.23 ± 0.1 GPa	−0.01 ± 0.005 GPa
Cu_2_	0.52 GPa	0.44 GPa	−0.01 GPa
Mo	−0.48 ± 0.2 GPa	−0.53 ± 0.2 GPa	−0.05 ± 0.01 GPa
