# Supplementary material for: Modified crystallite group method for residual stress analysis of highly textured Cu/Mo nanomultilayers
Source: J Appl Crystallogr. 2026 May 20;59(Pt 3):910–22. doi: 10.1107/S1600576726003638 (PMC13224809; doi:10.1107/S1600576726003638)
Supplement: Supplementary file 1 [file j-59-00910-sup1.pdf]

JOURNAL OF
APPLIED
CRYSTALLOGRAPHY

Volume 59 (2026)

Supporting information for article:

Modified crystallite group method for residual stress analysis of highly textured Cu/Mo nanomultilayers

Jeyun Yeom, Claudia Cancellieri, Amit Sharma, Bastian Rheingans, Xavier Maeder, Gregory Abadias and Jolanta Janczak-Rusch

Supplementary Materials

Modified crystallite group method for residual-stress analysis of highly textured Cu/Mo nano-multilayers

Jeyun Yeom [a)] [1] Claudia Cancellieri [a)1*] Amit Sharma[2] Bastian Rheingans[1] Xavier Maeder[2] Gregory Abadias[3] Jolanta Janczak-Rusch[1]

[1]Empa, Swiss Federal Laboratories for Materials Science and Technology, Laboratory for Joining Technologies and Corrosion, Überlandstrasse 129, 8600 Dübendorf, Switzerland

[2]Empa, Swiss Federal Laboratories for Materials Science and Technology, Laboratory for Mechanics of Materials & Nanostructures, Feuerwerkstrasse 39, Thun CH-3602, Switzerland

[3]Institut Pprime, Université de Poitiers-CNRS-ENSMA, Boulevard Marie et Pierre Curie, 86073 Poitiers Cedex 09, France

*claudia.cancellieri@empa.ch

[a)] Equal contributions

1. Microstructure of Cu/Mo NML

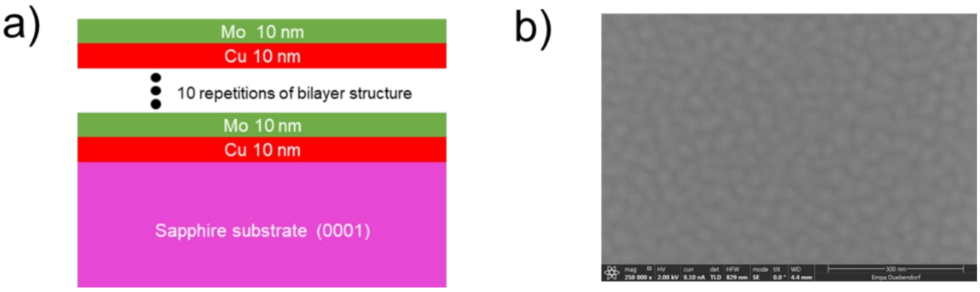

Figure S1. a) Illustration of Cu/Mo NML on sapphire (0001) substrate, SEM surface image of Cu/Mo NML

Figs. S1a-b display a schematic illustration of the Cu/Mo NML on sapphire (0001) substrate, along with SEM surface images of the as-prepared Cu/Mo NML structure. In this structure, uniformly formed grain-like structures were observed without any cracks or voids. Figs. S2a, b show the BF TEM and HAADF STEM cross-sectional images of a sample, respectively. In BF TEM images, alignment of a crystal close to a zone axis results in darker contrast. This suggests that defects inside the Cu/Mo NML contribute to a gradual change in contrast, and that it is not a perfect single crystal formed by epitaxial growth. Meanwhile, in the HAADF STEM image, the Cu and Mo layers exhibited bright and dark contrast, respectively, indicating that the alternating layers have clear periodic structure without significant voids. This fact can also be confirmed from the EDX mapping images presented in Figs. S2c, d. The deposited nanolaminate structure was initially flat and smooth, near the substrate interface.

However, with a further increase in thickness, as is clear in Fig. S1b, the structure became wavy, forming polycrystalline grain-like structures. Such wavy structures have also been observed in other NML systems, such as Cu/W (Moszner *et al.*, 2016; Monclús *et al.*, 2014; Druzhinin *et al.*, 2019), Cu/Mo (Srinivasan *et al.*, 2006), and Cu/Nb (Yeom *et al.*, 2023). These images suggest that the Cu/Mo NMLs exhibit a Cu_{10nm}-Mo_{10nm} bilayer structure with 10 repetitions, and that they were well prepared without any voids.

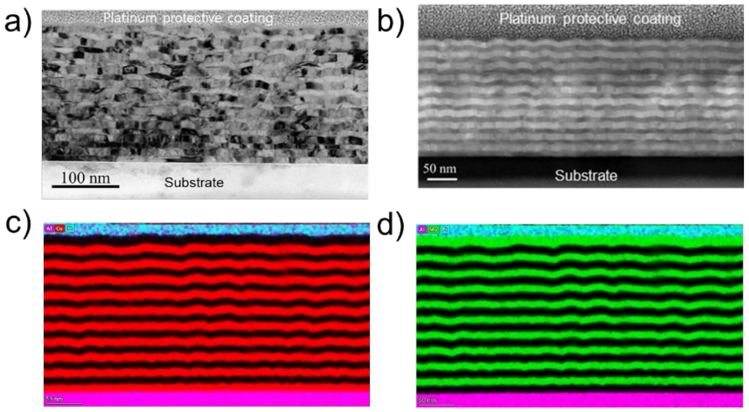

Figure S2. a) Cross-sectional BF TEM image of Cu/Mo NML, b) cross-sectional HAADF STEM image of Cu/Mo NML, c)-d) EDX mapping images of Cu/Mo NML.

2. Selected diffraction plane of Cu

2.1 Cu diffraction planes with three-fold rotational symmetry

For the evaluation of the residual stress of Cu layer in this sample, the following equation, which corresponds to Eqn. 19 in the main manuscript, should be adjusted depending on the choice of diffraction planes: $\epsilon_{33}^{L} = [\frac{1}{4}s_{44}(\sigma_{11}+\sigma_{22}) + \frac{1}{12}(3s_{44}+2s_0)\{(\sigma_{11}-\sigma_{22})\cos 2\Phi + 2\sigma_{12}\sin 2\Phi\}]\sin^2\Psi - \frac{\sqrt{2}}{6}s_0[(\sigma_{11}-\sigma_{22}) < \cos(3\beta-2\Phi) > -2\sigma_{12} < \sin(3\beta-2\Phi) >]\sin 2\Psi + \frac{1}{3}(3s_{12}+s_0)(\sigma_{11}+\sigma_{22})$ (S1). In the case of Cu diffraction planes with three-fold rotational symmetry, Eqn. S1 is transformed through the averaging process into $\epsilon_{33}^{L} = [\frac{1}{4}s_{44}(\sigma_{11}+\sigma_{22}) + \frac{1}{12}(3s_{44}+2s_0)\{(\sigma_{11}-\sigma_{22})\cos 2\Phi + 2\sigma_{12}\sin 2\Phi\}]\sin^2\Psi - \frac{\sqrt{2}}{6}s_0[(\sigma_{11}-\sigma_{22})(-\cos(2\Phi)) - 2\sigma_{12}(\sin(2\Phi))]\sin 2\Psi + \frac{1}{3}(3s_{12}+s_0)(\sigma_{11}+\sigma_{22})$ (S2).

For the points of $\Phi = -60\,°$ (see Fig. S3), Eqn. S2 changes into $\epsilon_{33}^{L} = [\frac{1}{4}s_{44}(\sigma_{11}+\sigma_{22}) + \frac{1}{12}(3s_{44}+2s_0)\{(\sigma_{11}-\sigma_{22})(-0.5) - \sqrt{3}\sigma_{12}\}]\sin^2\Psi - \frac{\sqrt{2}}{6}s_0[(\sigma_{11}-\sigma_{22})(0.5) + \sqrt{3}\sigma_{12}]\sin 2\Psi + \frac{1}{3}(3s_{12}+s_0)(\sigma_{11}+\sigma_{22})$ (S3). Likewise, for the points where $\Phi = 60°, -180°$ (see Fig. S3), the

Eqn. S2 changes into $\epsilon_{33}^{L} = [\frac{1}{4}s_{44}(\sigma_{11}+\sigma_{22}) + \frac{1}{12}(3s_{44}+2s_0)\{(\sigma_{11}-\sigma_{22})(-0.5) + \sqrt{3}\sigma_{12}\}]\sin^2\Psi - \frac{\sqrt{2}}{6}s_0[(\sigma_{11}-\sigma_{22})(0.5) - \sqrt{3}\sigma_{12}]\sin 2\Psi + \frac{1}{3}(3s_{12}+s_0)(\sigma_{11}+\sigma_{22})$ (S4) and $\epsilon_{33}^{L} = [\frac{1}{4}s_{44}(\sigma_{11}+\sigma_{22}) + \frac{1}{12}(3s_{44}+2s_0)(\sigma_{11}-\sigma_{22})]\sin^2\Psi + \frac{\sqrt{2}}{6}s_0[(\sigma_{11}-\sigma_{22})]\sin 2\Psi + \left(s_{12}+\frac{1}{3}s_0\right)(\sigma_{11}+\sigma_{22})$ (S5), respectively.

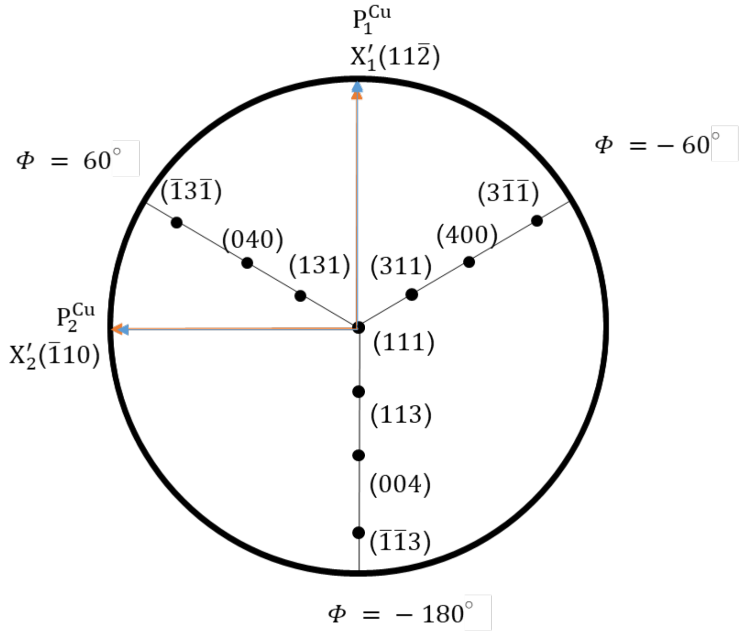

Figure S3. Schematic representations of crystallographic plane symmetries relevant to XRD reflections in cubic crystals, illustrated using stereographic projections (111) orientation, showing diffraction planes with three-fold rotational symmetry, used for measuring residual stress except (111) plane, where P and X' represent the coordinates of the specimen and transformed crystal, respectively.

2.2 Cu diffraction planes with six-fold rotational symmetry

As explained in the main manuscript, through the averaging process, Eqn. S1 is transformed into: $\epsilon_{33}^{L} = [\frac{1}{4}s_{44}(\sigma_{11}+\sigma_{22}) + \frac{1}{12}(3s_{44}+2s_0)\{(\sigma_{11}-\sigma_{22})\cos 2\Phi + 2\sigma_{12}\sin 2\Phi\}]\sin^2\Psi + \frac{1}{3}(3s_{12}+s_0)(\sigma_{11}+\sigma_{22})$ (S6).

For the points where $\Phi = -90°$(see Fig. S4), Eqn. S6 becomes $\epsilon_{33}^{L} = [\frac{1}{4}s_{44}(\sigma_{11}+\sigma_{22}) + \frac{1}{12}(3s_{44}+2s_0)\{(\sigma_{11}-\sigma_{22})(-1)\}]\sin^2\Psi + \frac{1}{3}(3s_{12}+s_0)(\sigma_{11}+\sigma_{22})$ (S7).

For the points where $\Phi = -150°, 150°$ (see Fig. S4), Eqn. S6 becomes: $\epsilon_{33}^{L} = \left[\frac{1}{4}s_{44}(\sigma_{11}+\sigma_{22}\right) + \frac{1}{12}(3s_{44}+2s_0)\{(\sigma_{11}-\sigma_{22})(0.5) + \sqrt{3}\sigma_{12}\}]\sin^2\Psi + \frac{1}{3}(3s_{12}+s_0)(\sigma_{11}+\sigma_{22})$ (S8),

$\epsilon_{33}^{L} = \Big[\frac{1}{4}s_{44}(\sigma_{11}+\sigma_{22}\Big) + \frac{1}{12}(3s_{44}+2s_0)\{(\sigma_{11}-\sigma_{22})(0.5) - \sqrt{3}\sigma_{12}\}]\sin^2\Psi + \frac{1}{3}(3s_{12} + s_0)(\sigma_{11}+\sigma_{22})$ (S9), respectively.

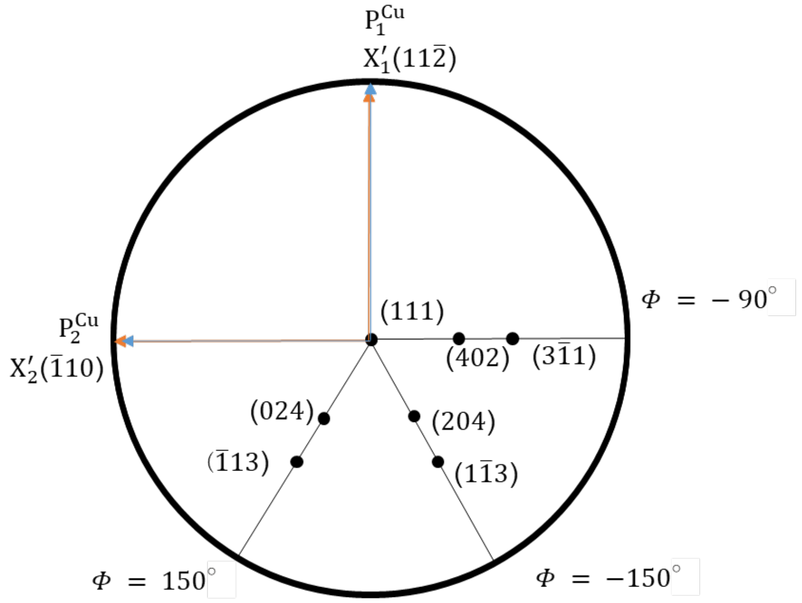

Figure S4. Schematic representations of crystallographic plane symmetries relevant to XRD reflections in cubic crystals, illustrated using stereographic projections in (111) orientation, showing diffraction planes with six-fold rotational symmetry used for measuring residual stress except (111) plane, where P and X' represent the coordinates of the specimen and transformed crystal, respectively.

3. Selected diffraction plane of Mo

As explained in the main manuscript, Eqn. 25 is modified depending on the choice of diffraction planes. For simplicity, Eqn. 25 in the main manuscript is given as:

$\epsilon_{33}^{L} = [\frac{1}{4}(s_{44}+\frac{1}{2}s_0)(\sigma_{11}+\sigma_{22}) + \frac{s_0}{8}(\sigma_{11}+\sigma_{22}) < \cos 2\beta > + \frac{4s_{44}+3s_0}{16}\{(\sigma_{11}-\sigma_{22})\cos 2\Phi + 2\sigma_{12}\sin 2\Phi\} + \frac{3s_0}{16}(\sigma_{11}-\sigma_{22})\{< \cos 2(2\beta-\Phi) > + 2 < \cos 2(\beta-\Phi) >\} - \frac{3}{8}s_0\sigma_{12}\{< \sin 2(2\beta-\Phi) > + 2 < \sin 2(\beta-\Phi) >\}]\sin^2\Psi - \frac{1}{4}s_0\{(\sigma_{11}-\sigma_{22}) < \cos 2(\beta-\Phi) > - 2\sigma_{12} < \sin 2(\beta-\Phi) >\} + \frac{1}{4}(4s_{12}+s_0)(\sigma_{11}+\sigma_{22})$ (S10).

For the points where $\Phi = -144.74°$ (see Fig. S5), Eqn. S10 is transformed into:

$\epsilon_{33}^{L} = [\frac{1}{4}(s_{44}+\frac{1}{2}s_0)(\sigma_{11}+\sigma_{22}) + \frac{s_0}{8}(\sigma_{11}+\sigma_{22})(0.3334) + \frac{4s_{44}+3s_0}{16}\{(\sigma_{11}-\sigma_{22})(0.3334) + 2 * (0.9427)\sigma_{12}\} + \frac{3s_0}{16}(\sigma_{11}-\sigma_{22})(2.3334) - \frac{3}{8}s_0\sigma_{12}(0.9427)]\sin^2\Psi - \frac{1}{4}s_0(\sigma_{11}-\sigma_{22}) + \frac{1}{4}(4s_{12} + s_0)(\sigma_{11}+\sigma_{22})$ (S11).

For the points where $\Phi = -35.26°$ (see Fig. S5), the averaging process is as follows:

$< \cos 2\beta > = \frac{1}{2}[\cos 2(-35.26°) + \cos 2(144.74°)] = 0.3334$ (S12),

$< \cos 2(2\beta - \Phi) > \; = \; \frac{1}{2}[\cos 2\{2(-35.26°) - (-35.26°)\} + \cos 2\{2(144.74°) - (-35.26°)\}] \; =$ 0.3334 (S13),

$< \cos 2(\beta - \Phi) > \; = \; \frac{1}{2}[\cos 2\{(-35.26°) - (-35.26°)\} + \cos 2\{(144.74°) - (-35.26°)\}] \; = \; 1$ (S14),

$< \sin 2(2\beta - \Phi) > \; = \; \frac{1}{2}[\sin 2\{2(-35.26°) - (-35.26°)\} + \sin 2\{2(144.74°) - (-35.26°)\}] \; =$ -0.9427 (S15),

$< \sin 2(\beta - \Phi) > \; = \; \frac{1}{2}[\sin 2\{(-35.26°) - (-35.26°)\} + \sin 2\{(144.74°) - (-35.26°)\}] \; =$ 0 (S16). For the evaluation of residual stress using these points, Eqn. S10 should be changed into $\epsilon_{33}^{L} \; = \; [\frac{1}{4}(s_{44} + \frac{1}{2}\, s_0)(\sigma_{11} + \sigma_{22}) + \frac{s_0}{8}\,(\sigma_{11} + \sigma_{22})(0.3334) + \frac{4s_{44} + 3\, s_0}{16}\{(\sigma_{11} - \sigma_{22})\,(0.3334) + 2\,(-0.9427)\sigma_{12}\} + \frac{3\, s_0}{16}(\sigma_{11} - \sigma_{22})(2.3334) + \frac{3}{8} s_0 \sigma_{12}(0.9427)]\, \sin^2\Psi - \frac{1}{4} s_0(\sigma_{11} - \sigma_{22}) + \frac{1}{4}(4s_{12} + s_0)(\sigma_{11} + \sigma_{22})$ (S17).

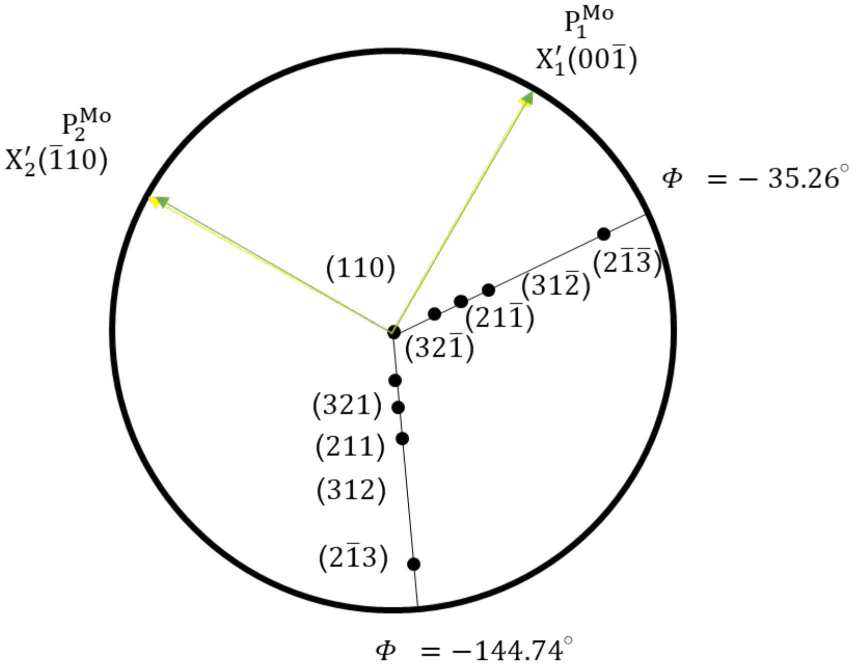

Figure S5. Schematic representations of crystallographic plane symmetries relevant to XRD reflections in cubic crystals, illustrated using stereographic projections in (110) orientation, showing diffraction planes used for measuring residual stress except (110) plane, where P and X' represent the coordinates of the specimen and transformed crystal, respectively.

4. Fitting procedure to extract the stress $\sigma_{i,j}$ components and uncertainty analysis

A. Least-squares formulation

The residual stress components were obtained from nonlinear regression of the strain–stress relations derived within the modified Crystallite Group Method (CGM). The fitting was performed using a weighted nonlinear least-squares approach.

For each measured diffraction condition i, the residual is defined as:

$$r_i = \frac{a_i^{\text{model}} - a_i^{\text{exp}}}{\sigma_{a,i}}$$

where:

- a_i^{exp} is the experimentally determined lattice parameter,
- a_i^{model} is the model value calculated from the stress–strain relations,
- $\sigma_{a,i}$ is the experimental uncertainty of the lattice parameter.

The objective function minimized is:

$$\chi^2 = \sum_i r_i^2$$

All fits were performed using nonlinear optimization (MATLAB, weighted residual formulation).

B. Experimental weighting

The uncertainty of each lattice-plane spacing was obtained from Gaussian fits of the diffraction peaks. The resulting uncertainty in lattice parameter determination was typically:

- $\sigma_a \leq 0.007$ Å(general upper bound),
- specific fits used the experimentally determined value per dataset (e.g., 0.005 Å or 0.002 Å as appropriate).

These uncertainties were used directly as weighting factors in the least-squares formulation.

Weighting ensures that:

- Points with smaller experimental uncertainty contribute more strongly to the solution.
- Reported stress uncertainties properly propagate measurement errors.

C. Parameter uncertainty estimation

Formal uncertainties of the fitted stress components were calculated from the covariance matrix of the least-squares problem.

The Jacobian matrix J of the weighted residual vector was evaluated numerically at the optimum:

$$J_{ij} = \frac{\partial r_i}{\partial p_j}$$

where p_j denotes the j-th fitted parameters in the parameter vector [a_0, σ_{11}, σ_{22}, σ_{12}]
The parameter covariance matrix was approximated as:

$$\mathrm{cov}(p) \approx s^2(J^\mathrm{T}J)^{-1}$$

where:

$$s^2 = \frac{\chi^2}{N - P}$$

with:

- N= number of data points,
- P= number of fitted parameters.

The reported uncertainties correspond to 95% confidence intervals, calculated as:

$$\Delta p_{95\%} = t_{0.975}(N - P)\sqrt{\mathrm{diag}(\mathrm{cov}(p))}$$

where the $t_{0.975}$ is the 97.5th percentile of the distribution: the quantile 0.975 is used because a two-sided 95% confidence interval leaves 2.5% probability in each tail of the *t*-distribution. The 95% confidence intervals of the fitted parameters were calculated as

$$p_j \pm t_{0.975,\nu}\, SE_j,$$

where $p_j$is the j-th fitted parameter, SE_jits standard error obtained from the diagonal elements of the covariance matrix, and $t_{0.975,\nu}$is the 97.5th percentile of the Student's *t*-distribution with $\nu = N - P$degrees of freedom (N being the number of data points and $P$the number of fitted parameters). The factor $t_{0.975,\nu}$accounts for the finite sample size of the nonlinear least-squares problem.
This approach accounts for parameter correlations inherent to the multivariate stress model.

D. Assessment of fitting quality

The goodness of fit was evaluated using the reduced chi-squared statistic:

$$\chi^2_{\mathrm{red}} = \frac{\chi^2}{N - P}$$

For all datasets and fitting configurations, the reduced chi-squared values lie in the range:

$$\chi^2_{\mathrm{red}} \approx 0.6 - 0.9$$

Values slightly below unity indicate that the experimentally estimated uncertainties—derived independently from peak-fit statistics—are mildly conservative. The results demonstrate that:

- The model reproduces the experimental data within the estimated measurement errors,
- No evidence of overfitting is present,

- The weighting scheme is internally consistent.

The fits for Mo and C_u2 data sets are reported in the Fig. S6 a) and b) respectively.

a) **b)**

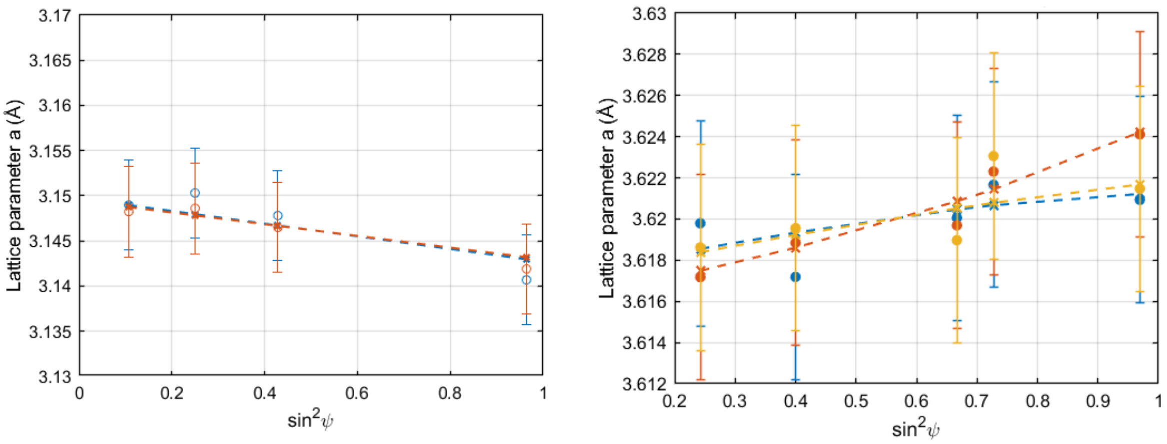

Figure S6: a) Experimental lattice parameters a derived from the selected diffraction groups are plotted as a function of $\sin^2\psi$ for a) Mo and b) Cu. Error bars correspond to the assumed experimental uncertainty of ±0.005 Å per data point, obtained from Gaussian fits of the diffraction peaks. The data are grouped by azimuthal angle φ; each color denotes one φ orientation, with symbols representing the experimental points. Dashed lines represent the nonlinear least-squares fit curves calculated from the best-fit stress tensor $(\sigma_{11}, \sigma_{22}, \sigma_{12})$ and stress-free lattice parameter a_0, which were refined simultaneously for all selected diffraction groups.

References

Druzhinin, A. V., Ariosa, D., Siol, S., Ott, N., Straumal, B. B., Janczak-Rusch, J., Jeurgens, L. P. H. & Cancellieri, C. (2019). *Materialia* **7**, 100400.

Monclús, M. A., Karlik, M., Callisti, M., Frutos, E., Llorca, J., Polcar, T. & Molina-Aldareguía, J. M. (2014). *Thin Solid Films* **571**, 275–282.

Moszner, F., Cancellieri, C., Becker, C., Chiodi, M., Janczak-Rusch, J. & P. H. Jeurgens, L. (2016). *J. Mater. Sci. Eng. B* **6**, 226–230.

Srinivasan, D., Sanyal, S., Corderman, R. & Subramanian, P. R. (2006). *Metall. Mater. Trans. A* **37**, 995–1003.

Yeom, J., Lorenzin, G., Cancellieri, C. & Janczak-Rusch, J. (2023). *Mater. Lett.* **352**, 135074.
